# Developing the eHistology Atlas

**DOI:** 10.1093/database/bav105

**Published:** 2015-10-24

**Authors:** Lorna Richardson, Liz Graham, Julie Moss, Nick Burton, Yogmatee Roochun, Chris Armit, Richard A. Baldock

**Affiliations:** MRC Human Genetics Unit, IGMM, University of Edinburgh, Edinburgh, UK

## Abstract

The eMouseAtlas project has undertaken to generate a new resource providing access to high-resolution colour images of the slides used in the renowned textbook ‘*The Atlas of Mouse Development*’ by Matthew H. Kaufman. The original histology slides were digitized, and the associated anatomy annotations captured for display in the new resource. These annotations were assigned to objects in the standard reference anatomy ontology, allowing the eHistology resource to be linked to other data resources including the Edinburgh Mouse Atlas Gene-Expression database (EMAGE) an the Mouse Genome Informatics (MGI) gene-expression database (GXD). The provision of the eHistology Atlas resource was assisted greatly by the expertise of the eMouseAtlas project in delivering large image datasets within a web environment, using IIP3D technology. This technology also permits future extensions to the resource through the addition of further layers of data and annotations to the resource.

**Database URL:** www.emouseatlas.org/emap/eHistology/index.php

## Introduction

*The Atlas of Mouse Development* by Matthew H. Kaufman (1) was published in 1992 and quickly established itself as the gold standard reference atlas for mouse developmental anatomy. The book comprises a temporal reference set of C57BL x CBA (F2) embryos throughout development from conception to birth, and in turn a series of histological sections for each embryo. These histology sections are annotated with labels detailing the various anatomical structures that can be identified at each stage. Despite the advancement of computational visualization software in the intervening years, and the availability of full 3D datasets (2–5), Kaufman’s ‘Atlas’ remains an important and much used reference in almost every mouse embryology lab.

While the printed atlas is still available, Elsevier hoped to have the original book brought up to date by the author with additional material included in response to a small survey of readers. This included additional images of coronal views particularly for neuroscience readers, and full-colour images for the histology plates. Sadly Professor Kaufman died before this was possible (6) and an alternative plan was developed, which was to produce an edited volume as a supplement to the atlas. This would bring the understanding of developmental anatomy up to date, system by system, and would include the new plate series of coronal sections. The first edition of this supplement is in press at the time of writing.

In parallel with the production of this new edition and supplement, the Mouse Atlas (eMouseAtlas.org) group in Edinburgh created a new digital resource providing free access to new high-resolution full-colour images of the histology sections used for the original book, via the eMouseAtlas resource. Through a close working relationship with Prof. Kaufman, the original haematoxylin and eosin stained slides were bequeathed to the eMouseAtlas project, and as such were available to be digitized for the purposes of this new resource. The availability of these new images, coupled with the expertise of the eMouseAtlas project in delivery of large image datasets across the web, enabled the provision of the eHistology Atlas (7).

## Design and Development of the eHistology Atlas

### IIP viewer

The eMouseAtlas IIP3D viewer employed for the display of the high-resolution sections was initially developed within the group (8) as a means of overcoming the issue of delivering very large tiled or 3D images within the constraints of a web browser. The combinations of operating systems and browsers for which the viewer is optimized are listed in the *browser compatibility* link. Using this technology for the eHistology Atlas will allow future extensions to the atlas to include additional markers (e.g. from the community), regional delineations (e.g. of anatomy) and potentially gene-expression overlays.

**Figure 1.**
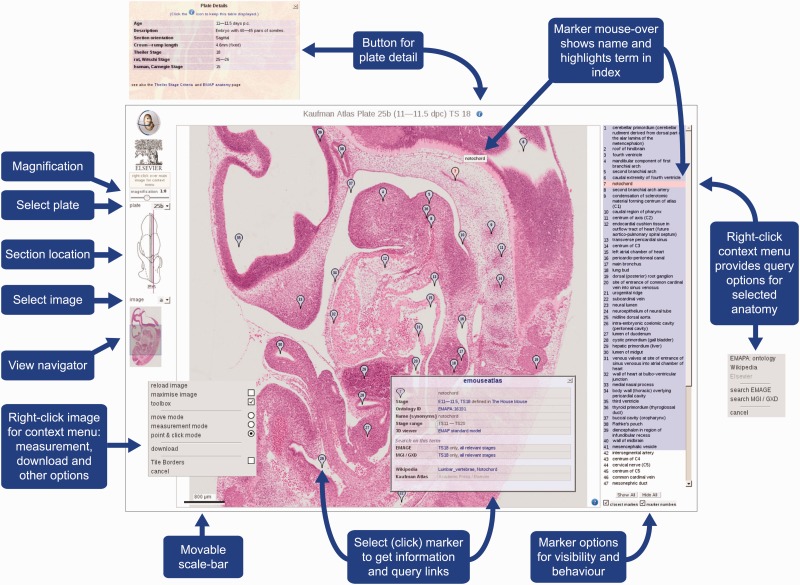
eHistology Atlas IIP3D Viewer Details. This figure shows the various options and navigation tools available within the IIP3D viewer used in the eHistology Atlas. Plate 25b (image a) is shown, with the colour section image displayed in the central panel at 1:8 magnification, the anatomy labels are listed on the right, and navigation tools shown on the left. Clicking on a numbered flag opens up the floating pop-up window containing internal and external links. Image is provided with permission from eMouseAtlas.

For the purposes of the eHistology Atlas, the layout of the IIP3D viewer has been adapted to suit the data (Figure 1). Users can move between any of the sections within a plate and then pan and zoom on this image. In parallel to the original book, the anatomy terms are listed in numbered order by plate. Selecting any of the terms in the list will highlight the flag labelling that structure if it was annotated on that image in the book. Alternatively, by hovering over the chosen section image, the three closest numbered flags to the cursor will appear, or the user can select to ‘show-all’ flags on the image. By clicking any chosen flag, a pop-up window appears with more information about the structure as well as the various links out to other resources described elsewhere in this text.

### Slide digitization and annotation

The starting point for the eHistology Atlas was the digitization of the original histology slides. This was accomplished using the Olympus DotSlide slide scanner system, which consists of an Olympus BX51 upright microscope equipped with an objective imaging motorized stage and an autonomous slide loader. Calibration was accomplished as part of the digitization process, allowing the inclusion of scale-bars, and the option to measure the distance between two points, as features of the eHistology Atlas. The specific sections included in the book were clearly marked on the glass slides by Kaufman, and only those 910 sections were scanned at high resolution. With respect to the total number of sections available in the Kaufman collection, this represented ∼10% of transverse sections, ∼5% of sagittal sections and <3% of coronal sections. The sections were scanned in full colour using a ×20 objective, resulting in a pixel resolution of 0.34 microns.

Despite the relatively long time (more than 20 years) between creation of the slides and this new digitization, the quality of the histological sections remained extremely good. The slides had been carefully stored in slide holders in the dark, and as such the staining did not suffer from substantial colour degradation. In the few cases where noticeable colour fading had occurred, this was confined to sections positioned at the very edges of the slide/coverslip. As the slide scanner was not able to capture these sections due to interference from the slide holder, in three cases a neighbouring section had to be used as a substitute.

It is noteworthy that in a few instances the original sections could not be sourced. In the case of Plate 5, only five of the ten sections were available for digitization, and in the case of Plate 14, none of the original sections could be sourced and were presumed lost. Fortunately, in each case the original photographic negatives were available and so could be used to generate cellular-resolution grey-scale images for display in the eHistology resource.

Having generated the high-resolution digital images, it was then necessary to add flags to indicate the position of the anatomical labels used in the book. By necessity, this was a manual process. Each numbered flag was positioned by eye on the correct region of the section according to the images in the book. The list of anatomical terms associated with each plate had been extracted using optical character recognition software, followed by a round of manual checking. Ultimately this process provided a list of numbered individual terms linked to the correct flags in position on the new images.

### Linking to the EMAP ontology

The Edinburgh Mouse Atlas Project (EMAP) anatomy ontology is considered to be the standard descriptive ontology for mouse developmental anatomy. It was developed originally by Bard *et al*. (9) and was based loosely on the anatomy terms listed in the index of Kaufman’s Atlas. It is structured as a set of timed partonomic ontologies, one for each Theiler stage (10), as well as an over-arching ‘abstract’ ontology that includes all terms from all stages. The ontology includes all visible anatomical structures linked by ‘part-of’ relationships. Since its development, the ontology has been improved and extended in line with the needs of the user community, principally through adding greater granularity of terms for specific substructures (11). Each term in the ontology has a unique ID, allowing other resources to link to and utilize the logic of the ontology for their own data. While the index of the Atlas was the original basis of the EMAP anatomy ontology, the labels used in the book were often very descriptive, and did not string match to the terms in the current ontology. To allow the eHistology Atlas to be integrated to informatic resources like EMAP, EMAGE and MGI, it was necessary for each flag label to be assigned an abstract EMAP ontology ID (EMAPA:). There are over 10 000 flag labels used to annotate the eHistology sections, and linking them to EMAPA IDs was achieved through a combination of string matching and manual assignment of terms. In the case of very descriptive labels, the actual term was often hidden within the text of the label. For example, the label ‘distal part of right ureter where it approaches the vesical part of the urogenital sinus’ would correctly map to EMAPA:17950 ‘ureter’. The process of assigning EMAPA IDs even guided the development of the ontology. For example, the term ‘adrenal gland’ which was previously not included in the ontology at TS21 was labelled on a TS21 section, thus indicating that it should be added to the ontology for that stage. This process of assigning new terms to the ontology was not a uniform decision but required curatorial input. Assigning each identified structure in the eHistology Atlas to an appropriate EMAPA ID, allows the eHistology Atlas interface to be a portal to relevant mouse data from distributed resources. Most notably the EMAPA IDs are used extensively within the EMAGE spatial gene expression database (12) and the MGI mouse genome informatics resource (13). By including EMAPA IDs, it is possible for a user to ask the question ‘what genes are expressed in this structure?’

### Additional links

In the interests of adding value to the resource, it was decided to include a variety of additional links from each structure, such as Wikipedia. While the very nature of Wikipedia dictates that the content of these links cannot be verified, in general it is the same end point that a user carrying out a web search will typically find. The process of matching EMAP and eHistology terms to Wikipedia pages was manually curated. A first pass for exact matches of terms to page titles was carried out. Where no exact match existed in Wikipedia (usually in the case of very specific structures), terms were then assigned to appropriate superstructures further up the ontology tree. For example, the terms ‘optic sulcus’ (EMAPA:16199) and ‘optic pit’ (EMAPA: 16325) refer to specific stages of eye development in the embryo. However, there are no direct Wikipedia pages for optic sulcus and optic pit, and so the terms link to the Wikipedia page for ‘eye development’ instead.

This eHistology resource was developed in collaboration with Academic Press/Elsevier who hold the copyright on the original Atlas. To allow the images, plate numbers, captions, caption numbering and layout to be made freely available, an agreement was made to allow Elsevier to also use the images in any future revision or online edition of the book (with text). We therefore have left available a link through to licenced material, such as the book text, for future purposes.

### Design of plate/stage browser

The intention was that the eHistology Atlas resource should complement not only the data, but also the organization of the book as far as possible, thus allowing it to function as a digital extension to the published text. For this reason, it was decided to retain the ‘plate-centric’ organization of the data images. In most cases, this correlates to a Theiler-stage centric view but for early stages, there may be more than one Theiler stage represented on a single plate.

**Figure 2.**
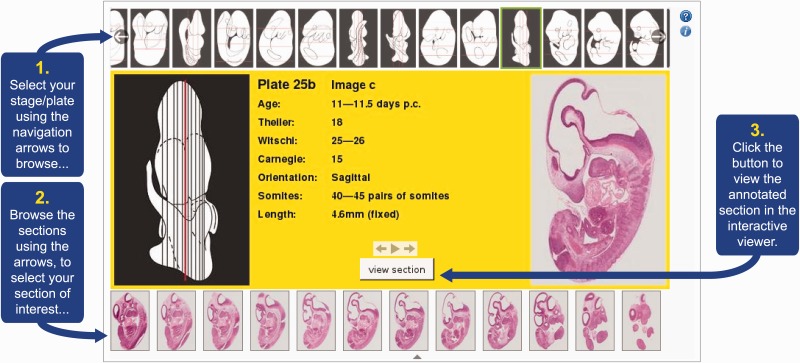
eHistology Atlas plate/stage browser. This figure shows the various elements of the eHistology Atlas plate/stage browser. Users are presented with this browser when they access the eHistology Atlas. They are able to scroll through the plates using the filmstrip browser at the top of the page. By selecting any particular plate, the user will then see the central panel in the browser alter to reflect the contents of the chosen plate. A larger version of the embryo schematic is shown on the left, with all of the section planes delineated to show the approximate position within the embryo, of each section. One section position is highlighted in red, and the relevant section image for this position is shown on the right. The user is able to change the selected section using the section filmstrip at the bottom of the page. The central panel displays the metadata for each selected plate. Having chosen a section of interest the user then launches the IIP3D viewer using the button in the central panel. Image is provided with permission from eMouseAtlas.

Users are able to browse the plates by way of a filmstrip display of embryo schematics at the top of the browser page (Figure 2). These schematics are designed to show the overall shape and form of an embryo, and importantly the orientation of the section plane by virtue of red lines superimposed on the illustration. Information on the age and developmental stage, as well as equivalent stage in other model organisms, is captured from the book and presented here. Any further information provided in the book is also included, e.g. embryo size (crown-rump length) and somite number are given where available. There is a further filmstrip along the bottom of the page allowing users to browse the sections relating to the plate selected. Finally the user can view the chosen section image in high-resolution in the specialized viewer (shown in Figure 1). They launch this viewer from a button in the central panel of the plate browser.

## Future Work

### Extension of eHistology resources

In developing the eHistology resource, we scanned the original sections that were used in Kaufman’s ‘*The Atlas of Mouse Development*’. This represents ∼10% of transverse sections, ∼5% of sagittal sections and <3% of coronal sections in the full Kaufman collection. Correspondingly, there is the possibility of extending the eHistology resource by including some, or all, of these intervening sections. For the benefit of the Deciphering the MEchanisms of Developmental Disorders (DMDD) project (3), we plan to generate additional atlas images of E14.5 sagittal sections, to support the phenotyping effort. Furthermore, and also in collaboration with the DMDD project, we aim to generate a placental histology atlas to supplement the eHistology embryo atlas. This placental atlas will consist of E9.5 and E14.5 placental histology images, with supporting anatomy annotations. With regards to annotation, it should be noted that while the EMAPA ontology currently includes some placental terms, in capturing expert annotation we plan to extend the EMAPA ontology where appropriate to include additional terms. This placental atlas will again assist in the phenotyping efforts of the DMDD as well as providing a valuable resource for other researchers with an interest in placentation.

### Query functionality

We plan to generate advanced query capabilities allowing users to filter for anatomical components of interest. In the most basic sense this would allow users to explore, e.g. images of the metanephros at all stages of development. A more advanced version would allow users to identify, e.g. all anatomical components that are known to develop from definitive endoderm. Identifying all components in this way would require an ontology that utilizes a *develops-from* relationship, allowing lineage of a tissue to be traced. It is noteworthy that an anatomical ontology with this structure has been described in the context of the developing human (14).

### Community resource/data tracks

As a community resource, we wish to introduce interfaces to enable community annotation of the eHistology Atlas. Towards this end, a ‘point-and-click’ interface was developed to allow invited experts to collaborate with the eMouseAtlas group in annotating a set of coronal sections generated by the late Professor Kaufman. This set of coronal sections will be included in a new Kaufman Atlas supplement that is currently in press. The ‘point-and-click’ interface allows multiple annotators to assign annotations simultaneously and thus represents the technical framework that is necessary for a community annotation interface. However, it will also be necessary to develop appropriate quality assurance methods before community annotation can be explored more thoroughly. In this respect, we envision that potential annotators will have to register as annotators, and all community annotations will be associated with a named annotator. Consequently, a user of the eHistology resource will be able to include or exclude external annotations. In addition to new annotation, we are interested in the possibility of using the eHistology Atlas as a community hub to link out to other data. Using the IIP3D technology that underlies the eHistology Atlas, users will be able to define points or regions in the atlas viewer, which are linked to data in an outside resource (via a url or doi). In effect, this would be analogous to ‘data-tracks’ that are a feature of genomic browsers such as Ensembl (15) and UCSC (16). 

## Funding

Funding for open access charge: Medical Research Council, UK, through core funding for the Mouse Atlas programme (grant number U.1275.2.4.4.1) and from the NIH/NIDDK (GUDMAP Project, NIH/NIDDK grant number DK092983).

*Conflict of interest*. None declared.

## References

[1] Kaufman M.H. (1992) The Atlas of Mouse Development, 1st edn Academic Press, New York.

[2] Armit C. Venkataraman S. Richardson L. (2012) eMouseAtlas, EMAGE, and the spatial dimension of the transcriptome. Mamm. Genome, 23, 514–524. 22847374 10.1007/s00335-012-9407-1 PMC3463796

[3] Mohun T. Adams D.J. Baldock (2013) Deciphering the Mechanisms of Developmental Disorders (DMDD): a new programme for phenotyping embryonic lethal mice, Disease Models and Mechanisms, 6, 562–566. doi:10.1242/dmm.011957 10.1242/dmm.011957 PMC3634640 23519034

[4] Dhenain M. Ruffins S.W. Jacobs R.E. (2001) Three-dimensional digital mouse atlas using high-resolution MRI. Dev. Biol., 232, 458–470. 11401405 10.1006/dbio.2001.0189

[5] Wong M.D. Dorr A.E. Walls J.R. (2012) A novel 3D mouse embryo atlas based on micro-CT. Development, 139, 3248–3256. 22872090 10.1242/dev.082016 PMC6514304

[6] Bard J. (2013) Matthew H. Kaufman (1942-2013) – mouse developmental anatomist. Development, 140, 4297–4298.

[7] Graham E. Moss J. Burton N. (2015) The atlas of mouse development eHistology resource. Development, 142, 1909–1911. 26015534 10.1242/dev.124917

[8] Husz Z. Burton N. Hill B. (2012) Web tools for large-scale 3D biological images and atlases. BMC Bioinformatics 13, 122. 22676296 10.1186/1471-2105-13-122 PMC3412715

[9] Bard J.L. Kaufman M.H. Dubreuil C. (1998) An internet-accessible database of mouse developmental anatomy based on a systematic nomenclature. Mech. Dev., 74, 111–120. 9651497 10.1016/s0925-4773(98)00069-0

[10] Theiler K. (1972) The House Mouse: Atlas of Embryonic Development, 1st edn Springer-Verlag, New York.

[11] Hayamizu T.F. Wicks M.N. Davidson D.R. (2013) EMAP/EMAPA ontology of mouse developmental anatomy: 2013 update. J. Biomed. Semantics, 4, 15. 23972281 10.1186/2041-1480-4-15 PMC3851555

[12] Richardson L. Venkataraman S. Stevenson P. (2014) EMAGE mouse embryo spatial gene expression database: (2014 update). Nucleic Acids Res., 42, D835–D844. 24265223 10.1093/nar/gkt1155 PMC3965061

[13] Smith C.M. Finger J.H. Hayamizu T.F. (2014) The mouse Gene Expression Database (GXD): 2014 update. Nucleic Acids Res., 42, D818–D824. 24163257 10.1093/nar/gkt954 PMC3965015

[14] Bard J. (2012) A new ontology (structured hierarchy) of human developmental anatomy for the first 7 weeks (Carnegie stages 1-20). J. Anat., 221, 406–416. 22973865 10.1111/j.1469-7580.2012.01566.x PMC3482348

[15] Cunningham F. Amode M.R. Barrell D. (2014) Ensembl 2015. Nucleic Acids Res., 43(Database issue):D662-9. doi: 10.1093/nar/gku1010. 10.1093/nar/gku1010 PMC4383879 25352552

[16] Mangan M.E. Williams J.M. Kuhn R.M. (2014) The UCSC Genome Browser: what every molecular biologist should know. Curr. Protoc. Mol. Biol., 107, 19.9.1–19.9.36. 24984850 10.1002/0471142727.mb1909s107 PMC4142428

